# Dietary Curcumin Alleviated Aflatoxin B1-Induced Acute Liver Damage in Ducks by Regulating NLRP3–Caspase-1 Signaling Pathways

**DOI:** 10.3390/foods10123086

**Published:** 2021-12-13

**Authors:** Sanjun Jin, Hao Yang, Yingjie Wang, Qian Pang, Yihan Jiao, Anshan Shan, Xingjun Feng

**Affiliations:** 1 Institute of Animal Nutrition, Northeast Agricultural University, Harbin 150030, China; Sanjunjin@163.com (S.J.); yanghao951209@163.com (H.Y.); wangyingjie@neau.edu.cn (Y.W.); pangqian1210@163.com (Q.P.); yihanjiao11@163.com (Y.J.); asshan@neau.edu.cn (A.S.); 2 Centre of Sport Nutrition and Health, Zhengzhou University, Zhengzhou 450001, China

**Keywords:** curcumin, acute liver, AFB1-DNA adducts, Nrf2-ARE, NLRP3–caspase-1

## Abstract

Aflatoxin B1 (AFB1) is a mycotoxin widely distributed in animal feed and human food; it represents a serious threat to human and animal health. This study investigates the mechanism by which dietary curcumin protected liver against acute damage caused by AFB1 administration in ducks. One-day-old male ducks (*n* = 450) were randomly assigned to three groups, the control group, the AFB1 group, and the AFB1 + curcumin group; the first group were fed with basic diet, while the third group was fed basic diet containing 500 mg/kg curcumin. Ducks in the AFB1 group and AFB1 + curcumin group were challenged with AFB1 at the age of 70 days. The results show that AFB1 administration caused liver damage, increased CYP450 content and AFB1-DNA adducts in the liver, and induced oxidative stress and inflammatory response in the liver. Dietary curcumin significantly inhibited the generation of H_2_O_2_ and MDA in liver, activated the Nrf2-ARE signaling pathway, and suppressed the NLRP3–caspase-1 signaling pathway in the liver of ducks. Conclusively, curcumin in diet could protect duck liver against the generation of AFB1-DNA adducts, toxicity, oxidation stress and inflammatory response induced by AFB1 through regulating the NLRP3–caspase-1 signaling pathways, demonstrating that curcumin is a potential feed additive agent to reduce the serious harmful effects of AFB1 on duck breeding.

## 1. Introduction

Aflatoxin B1 (AFB1), produced by *Aspergillus species*, is a stable toxic metabolite among the most toxic and carcinogenic metabolites [1]. AFB1 is as classified the most potent natural group 1 carcinogen by the International Agency for Research on Cancer (IARC) and resulted in 170,000 (28%) annual cases of human hepatocellular carcinoma cancer [2]. AFB1 can be found in poorly stored food or feeds, such as peanut, corn, rice, wheat, and soybean [3]. The consumption of food with AFB1 results in damage of the liver, as the liver is the target organ where the activation, metabolism, and elimination of toxins are all carried out [4,5,6]. In addition, AFB1 is mainly metabolized in the liver by cytochrome P450 (CYP450) bioactive enzymes including CYP1A1, CYP1A4, CYP2A6, and CYP3A4, contributing to disease development in the liver [7]. As well known, the pathophysiological processes of disease development are usually accompanied with oxidative stress and inflammation, such as the metabolic processes of AFB1 in the liver [8]. Excessive oxidation stress and inflammation play a vital role in the toxicity metabolism of AFB1 in the liver; as expected, antioxidants are an effective way to protect body against toxic metabolites, oxidative stress, and inflammation [9,10,11].

Curcumin is a kind of polyphenol component derived from the rhizome of turmeric (*Curcuma Longa* Linn.); it performs diverse biological activities, including serving as a free radical scavenger and antioxidant, as well as being vital in anti-inflammatory responses [12,13,14]. Studies reported that polyphenol component (resveratrol and curcumin) supplementation enhanced the antioxidant capacity of ducks by enhancing antioxidant enzyme activities [15,16]. Curcumin supplementation suppressed inflammation by inhibiting the activation of inflammasome in liver and kidney of mice and rats [17,18]. A research study reported the protective effects of curcumin on AFB1-induced liver damage by inhibition of the activity of CYP1A1 and AFB1-DNA adducts content in liver [19]. Pauletto et al. (2020) reported similar results, whereby, curcumin supplementation protected the liver of broilers against damage induced by AFB1 the inhibition of hepatic CYP2A6 gene expression [20]. Otherwise, curcumin has protective effects for the liver against damage induced by AFB1 by increasing the activity of antioxidant enzymes, e.g., by upregulating antioxidant genes expression in the nuclear factor erythroid 2-related factor 2 (NRF2) signaling pathway [5,21]. Furthermore, oxidation stress could activate the NLRP3 inflammasome and result in inflammation [22]. Pauletto et al. (2020) reported that curcumin supplementation inhibited inflammation by decreasing interleukin 1β (IL 1β) content in liver induced by AFB1 [20]. Therefore, curcumin supplementation has an ability to alleviate oxidation stress and inflammation induced by AFB1, but the application of curcumin as a feed additive for ducks to alleviate the damage of liver induced by AFB1 has not been researched.

This study established an antioxidant duck model; we investigate the metabolic mechanism of the protective effects of dietary curcumin on the acute liver damage induced by AFB1. This study provides a theoretical basis for the potential application of curcumin supplementation to protect avian health threatened by AFB1 administration.

## 2. Materials and Methods

### 2.1. Chemicals

Curcumin (CAS: 458-37-7) was obtained from Nanjing NutriHerb BioTech Co., Ltd. (Nanjing, China). AFB1 (CAS no. 1162-65-8) was obtained from Shanghai Yuanye Bio-Technology Co., Ltd. (Shanghai, China). Antibodies were obtained from Beyotime Biotechnology (Shanghai, China) including GAPDH (catalog number: AG019), caspase-1 (catalog number: AF1681), NLRP3 (catalog number: AF2155), horseradish peroxidase (HRP)-labeled Goat Anti-Mouse IgG (catalog number: A0216) and HRP-labeled Goat Anti-Rabbit IgG (catalog number: A0208).

### 2.2. Ducks and Husbandry

All ducks (*Anas platyrhynchos*) (*n* = 450) aged 1 day were randomly assigned to 3 groups (Table S1). Ducks were fed a corn–soybean basal diet formulated according to the National Research Council (1994) (Table S2) and 500 mg kg^−1^ curcumin was added in the basal diets for ducks in the T_500_ + AFB1 group. On the 70th days, ducks were fasted for 12 h and 15 were selected from each group, oral administration of phosphate-buffered saline (PBS) (T_0_), and of 60 μg of AFB1 kg^−1^ body weight (AFB1 was dissolved in PBS, for both T_0_ + AFB1 group and T_500_ + AFB1 group). All animal care and treatment regimens were performed in strict accordance with the regulation of the National Research Council Guide (1996) and Ethical and Animal Welfare Committee of Heilongjiang province, China (revised in 2016). The protocols employed in this study were approved by the Institutional Animal Care and Use Committee of Northeast Agricultural University (protocol number: Northeast Agricultural University (NEAU)-[2011]-9).

### 2.3. Sample Collection

Whole blood samples were obtained from duck wing veins 12 h after AFB1 administration and were then centrifuged (1000× *g* for 15 min at 4 °C) and stored at −80 °C. The liver was washed 3 times in ice-cold phosphate-buffered saline (PBS, Beyotime Biotechnology Shanghai, China; pH = 7.2–7.4), then immediately and individually stored at −80 °C for antioxidant enzymes activity and Real time quantitative PCR (qRT-PCR) analyses.

### 2.4. Histopathological Observation

About 0.125 cm^3^ of liver was quickly harvested and fixated with 4% paraformaldehyde for pathological studies. After paraffin embedding, the samples were cut and stained with hematoxylin and eosin (H&E) and observed with a light microscope (Nikon Eclipse Ci-L, Tokyo, Japan). The liver samples, at the level of 1 mm^3^, was fixed with 2.5% glutaraldehyde and 1% osmic acid, dehydrated and embedded in resin. A final examination using the transmission electron microscopy (TEM, H-7650, Hitachi, Tokyo, Japan) was performed after staining with uranyl acetate and lead citrate. 

### 2.5. Assay of CYP450 Content, AFB1-DNA Adducts Level and Antioxidant Ability in Liver

Liver samples were homogenized in a pre-cooled 0.9% stroke-physiological saline solution (4 °C, 0.9% NaCl, pH = 7.2–7.4) and centrifuged at 4 °C (5000× *g*, 10 min) to obtain the supernatant. The contents of CYP450 and AFB1-DNA adducts in the liver were determined by a competitive enzyme linked immune sorbent assay (ELISA) method, according to the manufacturer’s instructions (Nanjing Jiancheng Bioengineering Institute, Nanjing, China). The activity or content of total antioxidant capacity (T-AOC, U/mg protein), catalase (CAT, U/mg protein), total superoxide dismutase (T-SOD, U/mg protein), reductive glutathione glutathione S-transferase (GSH, μmol/mg protein), Glutathione S-transferase (GST, U/mg protein), hydrogen peroxide (H_2_O_2_, mmol/mg protein), and hydrogen peroxide (MDA, nmol/mg protein) of liver homogenates was measured using commercial kits (Nanjing Jiancheng Bioengineering Institute, Nanjing, China) according to the manufacturer’s instructions. 

### 2.6. Plasma Biochemical Assay

Hematological and biochemical parameters were determined using an automatic biochemical analyzer. The content or activity of total protein (TP, g/L), albumin (ALB, g/L), globulin (GLB, g/L), ALB/GLB (A/G), total bilirubin (TBIL, μmol/L), alkaline phosphatase (ALP, U/L), ALT (alanine aminotransferase, U/L), AST (alanine aminotransferase, U/L), and AST/ALT in the plasma was assessed with commercial kits (Nanjing Jiancheng Bioengineering Institute, Nanjing, China), according to the manufacturer’s guidelines.

### 2.7. RNA Isolation and Real-Time Quantitative Polymerase Chain Reaction (qRT-PCR)

Total RNA from frozen liver tissues was isolated utilizing TRIzol Reagent (TaKaRa, Dalian, China) according to Xue et al. (2021) with minor changes [23]. Total RNA concentration and purity was examined with a spectrophotometer (Implen NanoPhotometer P-330, Munich, Germany). Samples with an A260/A280 ratio between 1.8 and 2.0 were considered acceptable for the quality and integrity. In total, 1000 ng of total RNA was converted into cDNA using a Prime Script™ RT reagent kit containing gDNA Eraser (TaKaRa, Dalian, China). The obtained cDNA was amplified using a TB Green™ Premix Ex Taq™ (TaKaRa, Dalian, China) RT-PCR (qRT-PCR) kit. All PCR primers were obtained from NCBI and synthesized by Sangon Biotech Co., Ltd. (Shanghai, China) (Table S3). The target genes expression was determined by an ABI 7500 real-time PCR instrument (Perkin-Elmer, Applied Biosystems, Foster City, CA, USA). 

### 2.8. Western Blotting

Total protein of the liver was obtained and measured using an radioimmunoprecipitation (RIPA) buffer including PMSF (1 mmol/L) (Beyotime, Shanghai, China) and a BCA assay kit (Beyotime, Shanghai, China), respectively. Protein extracts were mixed with loading buffer and fully denatured in a boiling water bath according to Yang et al. (2021) with minor changes [24]. Target proteins were subjected to 8–12% SDS-PAGE electrophoresis, and transferred to a polyvinylidene-difluoride (PVDF) membrane (Beyotime, Shanghai, China). Afterwards, PVDF membranes were washed (3 times × 10 min in 1 × PBST), after blocking 2 h in 5% skim milk. After washing 3 times, PVDF membranes were incubated in primary antibody (GAPDH, NLRP3, and caspase-1) for 8–12 h at 4 °C. Related horseradish peroxidase labeled antibody was incubated at 37 °C for 1 h, after washing 3 times in PBS–0.1% Tween 20 (PBST). Protein bands were quantified and were recorded using the Essential V6 imaging platform (UVITEC, Cambridge, England) with the enhanced chemiluminescence (ECL) chemiluminescence kit (Beyotime Biotechnology). The GAPDH protein served as an internal control protein. The protein expression was expressed as the ratio of band intensities of proteins to that of GAPDH. 

### 2.9. Statistical Analysis 

The data were obtained and analyzed using the Independent-Sample *t*-Test in SPSS (Version 22.0, SPSS Inc., Chicago, IL, USA) with a 5% probability of error (*p* < 0.05). Graphs with standard error of the mean were plotted in GraphPad Prism 8.3.0 (GraphPad Software, San Diego, CA, USA) and formatted in Photoshop 2020CC (Adobe Systems, San Jose, CA, USA). 

## 3. Results 

### 3.1. Biochemical Levels in Plasma

**Figure 1:**
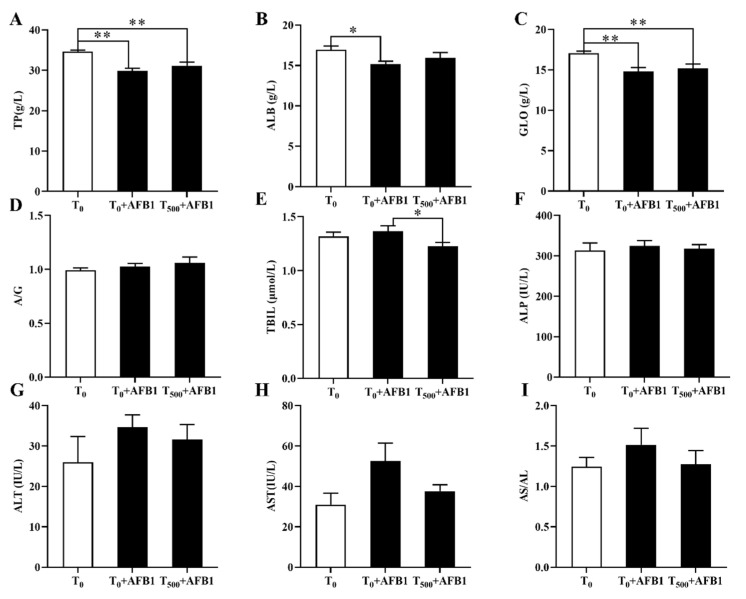
The plasma biochemical levels of ducks exposed to AFB1 at 12 h. (**A**) The TP content in the plasma of ducks; (**B**) The ALB content in the plasma of ducks; (**C**) The GLO content in the plasma of ducks; (**D**) The rate of ALB/GLO; (**E**) The TBIL activity in the plasma of ducks; (**F**) The ALP activity in the plasma of ducks; (**G**) The ALT activity in the plasma of ducks; (**H**) The AST activity in the plasma of ducks; (**I**) The rate of AST/ALT. Values mean the mean ± SEM (standard error (SE) of means.), * means *p* < 0.05, ** means *p* < 0.01.

Plasma biochemical indexes containing TP, ALB, GLO, TBIL, ALP, ALT, and AST were considered clinical signs in injured liver (Figure 1). We identified a significant decrease in TP (*p* < 0.01), ALB (*p* < 0.002) and GLO (*p* < 0.002) levels in the T_0_ + AFB1 group relative to those in the T_0_ group; however, there was no significant increase in TP (*p* = 0.262), ALB (*p* = 0.305), and GLO (*p* = 0.611) levels in the T_500_ + AFB1 group relative to those in the T_0_ + AFB1 group (Figure 1A–C). In comparison with the T_0_ group, a significant difference in TP (*p* < 0.01) and GLO (*p* < 0.01) levels in the T_500_ + AFB1 group was found; however, there was no significant difference in the ALB (*p* > 0.05) value. AFB1 administration increased TBIL content (*p* = 0.451) more in the T_0_ + AFB1 group than in the T_0_ group. Adding curcumin in diet significantly decreased TBIL level (*p* = 0.043) in the T_500_ + AFB1 group with respect to the T_0_ + AFB1 group. As expected, there was no significant difference in TBIL level between the T_500_ + AFB1 group and T_0_ group (*p* > 0.05) (Figure 1E). No significant difference in ALP (*p* = 0.621) and a decreasing trend in ALP (*p* = 0.676) were observed among groups (Figure 1F). There was no significant increase in ALT (*p* = 0.246) and AST (*p* = 0.065) activity in the T_0_ + AFB1 group relative to those in the T_0_ group. Adding curcumin into diet inhibited the activities of ALT (*p* = 0.544) and AST (*p* = 0.140) in the T_500_ + AFB1 group relative to those in the T_0_ + AFB1 group, but with no significant differences. No significant difference in ALT and AST activity between the T_0_ + AFB1 group and the T_0_ group was found (*p* > 0.05) (Figure 1G,H).

### 3.2. Evaluation of Pathological Sections and Ultrastructural Assessment in Liver

**Figure 2:**
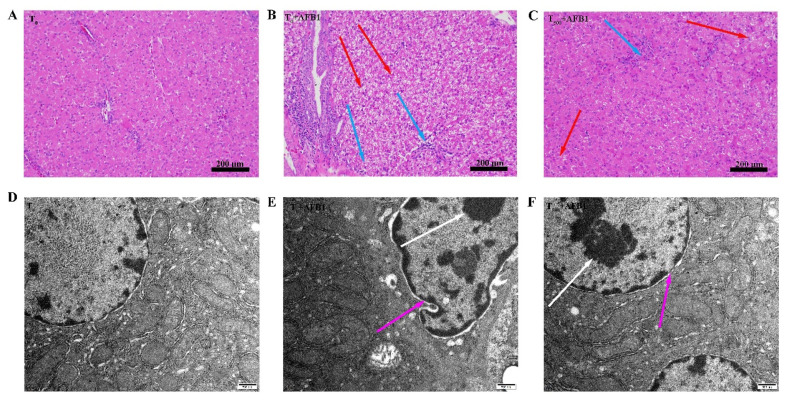
Histopathological and ultrastructure examination in liver of ducks exposed to AFB1 at 12 h. (**A**): control group (T_0_), (**B**): AFB1 group (T_0_ + AFB1); (**C**): curcumin + AFB1 group (T_500_ + AFB1); (**D**): control group (T_0_), (**E**): AFB1 group (T_0_ + AFB1); (**F**): curcumin + AFB1 group (T_500_ + AFB1). The blue arrowheads indicate swollen of liver cells, the red arrowheads indicate inflammatory cell infiltration, the white arrowheads indicate chromatin aggregation, and the pink arrowheads indicate the morphology of the cell nucleus.

Histopathological examination of H&E-stained livers shown in Figure 2. In the T_0_ group, hepatocytes morphology was normal (Figure 2A). AFB1 administration caused obvious toxicity containing vacuolation of hepatocytes, swelling of hepatocytes, and inflammatory cell infiltration in the T_0_ + AFB1 group compared to the T_0_ group (Figure 2B). Dietary curcumin protected the liver against damage through the decrease in the number of inflammatory cells and swelling of hepatocytes in the liver of ducks in the T_500_ + AFB1 group compared with in the T_0_ + AFB1 group (Figure 2C). A few inflammatory cells and swelling of hepatocytes in the T_500_ + AFB1 group compared with the T_0_ group was noticed. The results of this study demonstrate that dietary curcumin could protect duck liver against acute damage induced by AFB1 administration. The liver ultrastructure is shown in Figure 2. In the T_0_ group, the cell nucleus and mitochondrial ridge of hepatocytes were clearly visible and the chromatin in the cell nucleus was evenly distributed (Figure 2D). In comparison with the T_0_ group, the hepatocyte nucleus was visibly deformed; chromatin was aggregated and the hepatocyte mitochondrial ridge was enlarged and deformed in the T_0_ + AFB1 group (Figure 2E). As expected, in comparison with the T_0_ + AFB1 group, hepatocyte nucleus and mitochondrial ridge were clearly visible and the chromatin aggregation of hepatocytes was observed in the T_500_ + AFB1 group (Figure 2F). In addition, the hepatocyte nucleus and mitochondrial ridge were clearly visible when comparing the T_500_ + AFB1 group and T_0_ group. 

### 3.3. CYP450 Content in Liver

**Figure 3:**
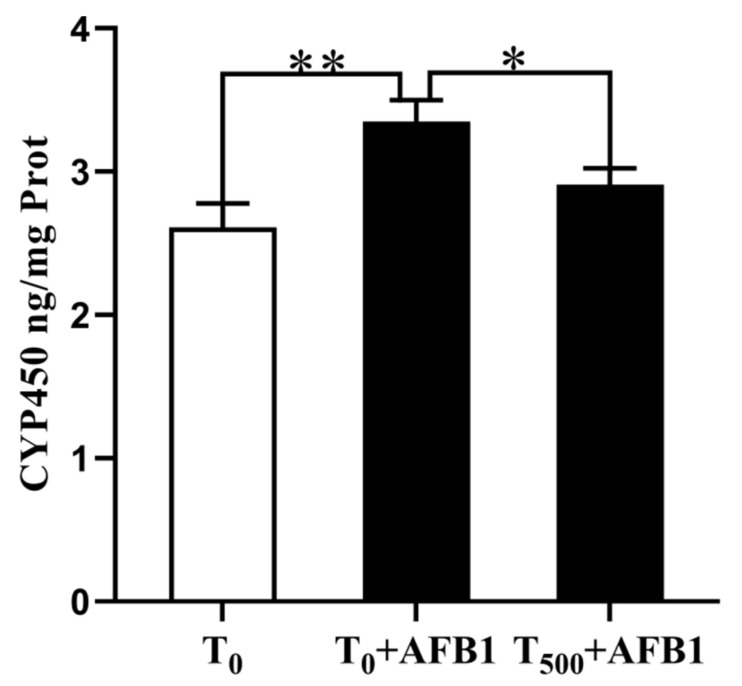
CYP450 level in liver of ducks exposed to AFB1 at 12 h. Values mean the mean ± SEM, * means *p* < 0.05, ** means *p* < 0.01.

Changes in CYP450 content in 10% liver homogenate are shown in Figure 3. There was a significant increase in CYP450 (*p* = 0.008) content in the T_0_ + AFB1 group relative to that in the T_0_ group. Dietary curcumin supplementation significantly attenuated the CYP450 aggregation (*p* = 0.041) in the liver of ducks in the T_500_ + AFB1 group compared with those in that in the T_0_ + AFB1 group. In addition, there was no significant increase in CYP450 aggregation between the T_500_ + AFB1 group and T_0_ group (*p* > 0.05).

### 3.4. AFB1-DNA Adducts in Liver

**Figure 4:**
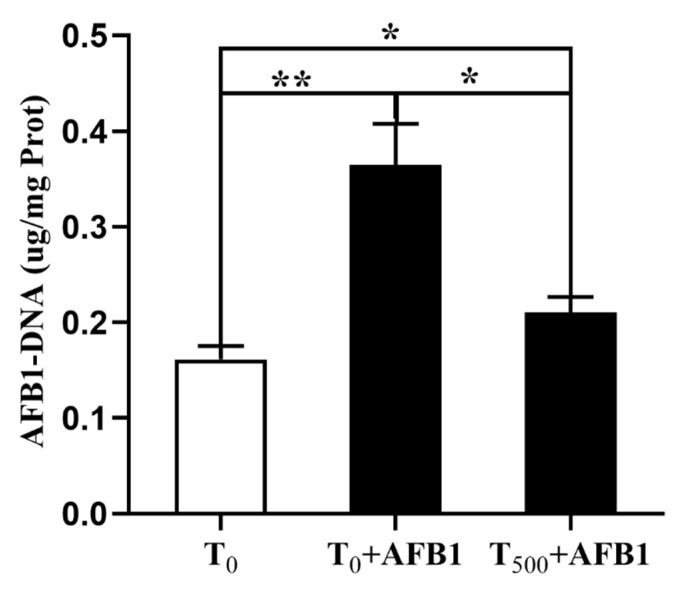
The AFB1-DNA adducts aggregation in liver of ducks exposed to AFB1 at 12 h. Values mean the mean ± SEM, * means *p* < 0.05, ** means *p* < 0.01.

The aggregation of AFB1-DNA adducts in liver is depicted in Figure 4. AFB1 administration significantly increased AFB1-DNA adducts in the liver (*p* = 0.004) of ducks in the T_0_ + AFB1 group compared with the T_0_ group. In addition, a significant decrease in AFB1-DNA adducts in the liver of ducks in the T_500_ + AFB1 group (*p* = 0.013) compared with the T_0_ + AFB1 group was found. Furthermore, a significant increase in AFB1-DNA adducts in the T_500_ + AFB1 group compared with the T_0_ group was observed (*p* < 0.05). 

### 3.5. Expression of Phase (I) Metabolic Enzyme Related Genes

**Figure 5:**
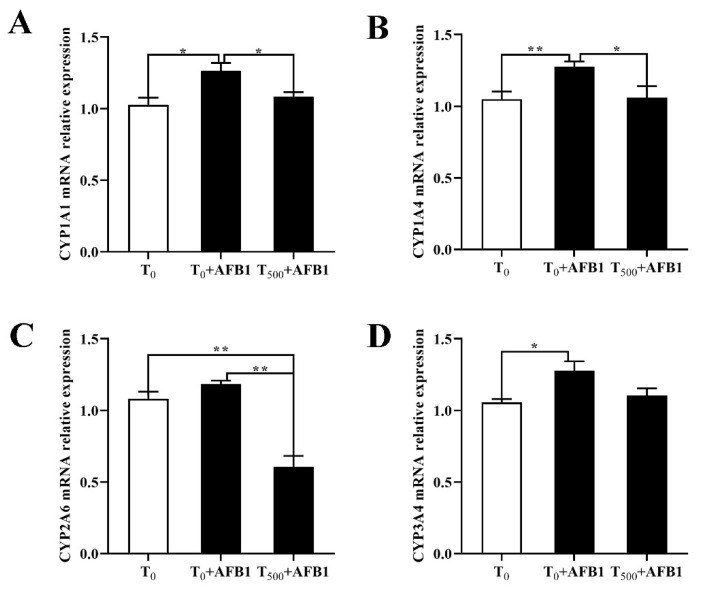
Expression of related phase (I) metabolic enzyme genes in the livers of ducks exposed to AFB1 at 12 h. (**A**) CYP1A1 gene expression in the liver of ducks; (**B**) CYP1A4 gene expression in the liver of ducks; (**C**) CYP2A6 gene expression in the liver of ducks; (**D**) CYP3A4 gene expression in the liver of ducks. Values mean the mean ± SEM, * means *p* < 0.05, ** means *p* < 0.01.

To investigate the role of the phase (I) metabolic enzymes in the attenuating effects of dietary curcumin on acute liver damage in ducks caused by AFB1 administration, the expression levels of genes related to the phase (I) metabolic enzymes (CYP1A1, CYP2A6, CYP1A4, and CYP3A4) were determined. As shown in Figure 5, AFB1 administration significantly increased mRNA levels of genes containing CYP1A1, CYP1A4, and CYP3A4 and showed an increasing trend of the CYP2A6 mRNA level in the liver of ducks in the T_0_ + AFB1 group compared with the T_0_ group. As predicted, adding curcumin into diet significantly decreased phase (I) metabolic enzymes genes expression, including the mRNA levels of CYP1A1, CYP1A4, and CYP2A6, in the liver of ducks in T_500_ + AFB1 group relative to those in the T_0_ + AFB1 group. In addition, it had no significant impact on the increase in mRNA expression including CYP1A1, CYP1A4, and CYP3A4 in the T_500_ + AFB1 group compared with the T_0_ group (*p* > 0.05) and showed a significant decrease in CYP2A6 gene expression. 

### 3.6. Antioxidant Capacity in Liver

**Figure 6:**
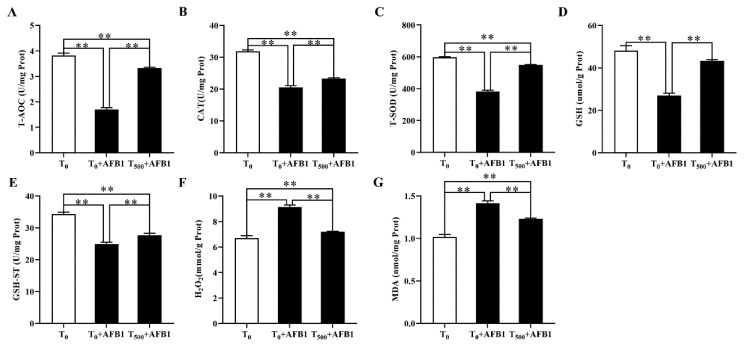
The antioxidant capacity in liver of ducks exposed to AFB1 at 12 h. (**A**) T-AOC activity in the liver of ducks; (**B**) CAT activity in the liver of ducks; (**C**) T-SOD activity in the liver of ducks; (**D**) GSH content in the liver of ducks; (**E**) GSH-ST activity in the liver of ducks; (**F**) H_2_O_2_ content in the liver of ducks; (**G**) MDA content in the liver of ducks. Values mean the mean ± SEM, ** means *p* < 0.01.

The antioxidant capacity in liver homogenate of ducks was demonstrated in Figure 6. In comparison with the T_0_ group, AFB1 administration significantly decreased T-AOC (*p* < 0.001), CAT (*p* < 0.001), T-SOD (*p* < 0.001), and GSH-ST (*p* < 0.001) activities and GSH (*p* < 0.001) content; it also increased H_2_O_2_ and MDA contents in the liver of ducks in the T_0_ + AFB1 group. Compared with the T_0_ + AFB1 group, dietary curcumin significantly increased the T-AOC (*p* < 0.001), CAT (*p* = 0.001), SOD (*p* < 0.001) and GSH-ST (*p* = 0.011) activities and GSH (*p* < 0.001) content in liver; further, it decreased of H_2_O_2_ and MDA contents in the liver. In addition, in comparison with the T_0_ group, a significantly decrease in the antioxidant enzyme activities of T-AOC (*p* < 0.01), CAT (*p* < 0.01), T-SOD (*p* < 0.01), GSH-ST (*p* < 0.01), and GSH (*p* < 0.001) was observed, along with a significant increase in the contents of H2O2 (*p* < 0.01) and MDA (*p* < 0.01) in the T_500_ + AFB1 group.

### 3.7. Expression of Molecules in the Nrf2-ARE Signaling Pathway

**Figure 7:**
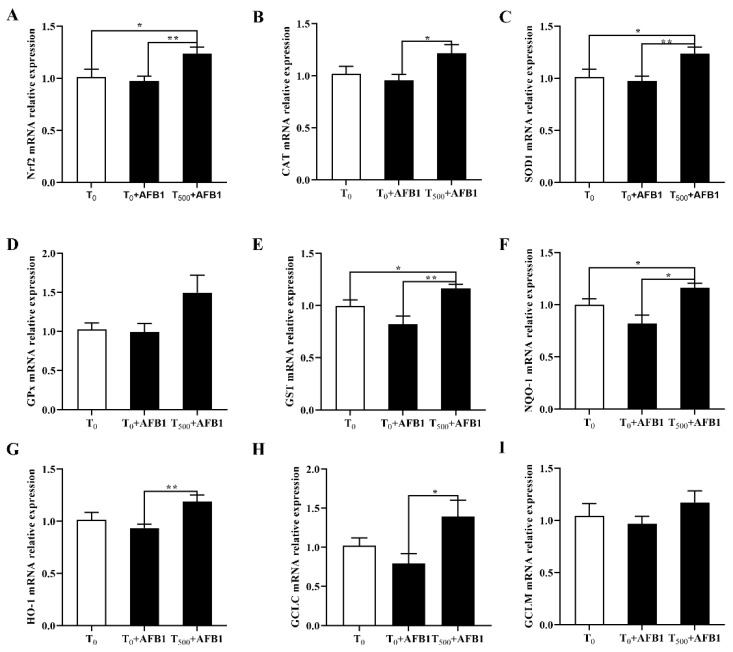
Gene expression in Nrf2 signaling pathway in liver of ducks exposed to AFB1 at 12 h. (**A**) Nrf2 gene expression in the liver of ducks; (**B**) CAT gene expression in the liver of ducks; (**C**) SOD1 gene expression in the liver of ducks; (**D**) GPx gene expression in the liver of ducks; (**E**) GST gene expression in the liver of ducks; (**F**) NQO-1 gene expression in the liver of ducks; (**G**) HO-1 gene expression in the liver of ducks; (**H**) GCLC gene expression in the liver of ducks; (**I**) GCLM gene expression in the liver of ducks. Values mean the mean ± SEM, * means *p* < 0.05, ** means *p* < 0.01.

AFB1 administration is associated with cell antioxidant response, suggesting the expression changes in genes participating in the Nrf2-ARE signaling pathway. As shown in Figure 7A, compared with the T_0_ group, AFB1 administration decreased the related genes expression including Nrf2 (*p* > 0.05), as well as enzymatic antioxidant system (CAT, SOD1, GPX1, and GST; *p* > 0.05) and phase (II) detoxifying enzymes (NQO1, HO-1, GCLC, GCLM; *p* > 0.05) in the T_0_ + AFB1 group. As expected, in comparison with the T_0_ + AFB1 group, curcumin supplementation significantly increased the genes expression levels including Nrf2, CAT, SOD1, GST, NQO1, HO-1, GCLC (*p* < 0.05) in the T_500_ + AFB1 group; further there was an increasing trend in the GPX1 and GCLM genes (*p* > 0.05). In addition, in comparison with the T_0_ group, a significant increase in genes expression of Nrf2, SOD1 and NQO1 (*p* < 0.05) and a generally increasing trend of gene expression of CAT, GST, HO-1, GCLC, GPX1, and GCLM (*p* > 0.05) in the T_0_ + AFB1 group were observed.

### 3.8. Expression of Molecules in the NLRP3–Caspase-1 Signaling Pathway

**Figure 8:**
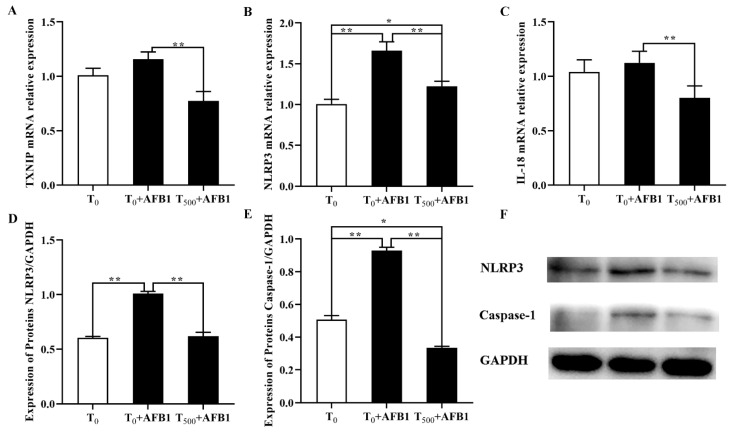
Expression of molecules in NLRP3–caspase-1 signaling way in liver of ducks exposed to AFB1 at 12 h. (**A**) TXNIP gene expression in the liver of ducks; (**B**) NLRP3 gene expression in the liver of ducks; (**C**) IL-18 gene expression in the liver of ducks; (**D**) NLRP3 protein expression in the liver of ducks; (**E**) NLRP3 protein expression in the liver of ducks; (**F**) Caspase-1 protein expression in the liver of ducks. Values mean the mean ± SEM, * means *p* < 0.05, ** means *p* < 0.01.

As shown in Figure 8, compared with the T_0_ group, the mRNA levels of NLRP3 in the liver of ducks were significantly increased after AFB1 administration and an increasing trend of TXNIP and IL-18 gene expression was observed in the T_0_ + AFB1 group. As expected, in comparison with the T_0_ + AFB1 group, dietary curcumin significantly decreased the mRNA levels of the genes NLRP3, TXNIP and IL-18 (*p* < 0.01) in liver of ducks in the T_500_ + AFB1 group (Figure 8A–C). In addition, compared to the T_0_ group, there was no significant decrease in the gene expression of NLRP3 and IL-18 (*p* > 0.05); however, there was a significant increase in TXNIP (*p* < 0.05) gene expression in the T_500_ + AFB1 group. the protein levels of NLRP3 and caspase-1 were significantly increased in the liver of ducks in the T_0_ + AFB1 group compared with the T_0_ group; whereas, compared to the T_0_ + AFB1 group, it had a significant impact on the decrease in NLRP3 and caspase-1 protein expression in the T_500_ + AFB1 group (Figure 8D–F). A significant decrease in caspase-1 protein expression (*p* < 0.05) was observed in the T_500_ + AFB1 group compared with the T_0_ group; whereas, there was a generally increasing trend in NLRP3 protein expression (*p* > 0.05). These results indicated that dietary curcumin protected the liver against injury induced by AFB1 administration by inhibiting the NLRP3–caspase-1 signaling pathway.

## 4. Discussion

In this study, blood metabolism disorders and abnormal liver enzyme activity occurred when animals were challenged with AFB1 administration. Abnormal metabolism, downregulation of TP, ALB and GLB and upregulation of metabolic enzyme activities (TBIL, ALP, ALT, and AST) in the plasma have been reported as indicators for liver toxicity [25]. Hematological biochemical disorders were reported in broilers when AFB1 was added into their diet (74 μg kg^−1^) [26]. Herein, a decrease in TP, ALB and GLB content and increases in TBIL content and activities of ALP, ALT, and AST in the plasma were caused by AFB1 administration; the blood metabolism disorders demonstrated that AFB1 led to serious liver damage in ducks. Similar results were reported by Wang et al. (2019), whereby AFB1 administration (40 μg/kg in the total mixed ration) increased the activity of ALB and GLO and increased TP content in the serum of dairy cows [27]. Meanwhile, dietary curcumin protected the liver against injury induced by AFB1 administration in this study. Li et al. (2019) reported that curcumin supplementation significantly slowed the increase in ALT and AST activity and the damage of liver induced by AFB1 [28]. In addition, our results indicate that adding curcumin into the diet protected duck liver against acute damage caused by AFB1 administration, which is consistent with the previous studies [7,19]. The blood metabolism disorders were also reflected the changes in liver morphology.

The liver is a vital detoxification organ in the body and the main target organ of AFB1 [29]. AFB1-contaminated diet induced liver damage as well as liver oxidation, mainly manifesting as inflammatory cell infiltration [10]. In this study, results of H&E staining and SEM demonstrate that morphological changes occurred in the liver of ducks after AFB1 administration, including enlargement and injury of hepatocellular tissues, inflammatory cell infiltration, and nuclear vacuolation and necrosis. We observed changes in the morphology and structure of hepatocytes induced by AFB1 administration indicating liver functional disorders, while adding curcumin into diet showed remarkable protective effects against histological toxin-induced injuries by AFB1 administration. In addition, little inflammatory cell infiltration and nuclear vacuolation and necrosis were observed in the T_500_ + AFB1 group compared with the T_0_ group. Furthermore, for rats, acute oral AFB1 (44–663 μg of AFB1 kg^−1^ of b. w.) led to liver damage, manifesting in inflammatory infiltrate, nuclear vacuolation and necrosis, in line with our results [30]. Similar results were reported for Cobb broilers, in which AFB1 induced histopathological lesions; grape seed proanthocyanidin extract (250 and 500 mg kg^−1^) + AFB1 (1 mg kg^−1^) mitigated AFB1’s negative effects in rats with sitagliptin activating the Nrf2-ARE-HO-1 signaling pathway to protect liver against AFB1-induced injury, while tea polyphenols protected hepatotoxicity against AFB1-induced injury in rats [29,30,31].

Synthesizing and enriching AFB1-DNA adducts in the liver by the activation of AFB1 in damaged liver morphology resulted in carcinogenic development [32]. After AFB1 administration, AFB1 is metabolized by cytochrome P450s isoenzymes to AFB1-8,9-epoxide (AFBO) and related adducts [33], which are aggregated in liver damage and oxidative DNA damage by ROS [34]. Therefore, the inhibition of AFB1-DNA adduct generation in liver would protects the liver against damage induced by AFB1. In this study, AFB1 administration significantly increased AFB1-DNA adducts in the liver; notably, there was a significant decrease in AFB1-DNA adducts in liver in the T_500_ + AFB1 group was observed, compared with the T_0_ + AFB1 group. No significant increase of the generation of AFB1-DNA adducts in the T_500_ + AFB1 group than that in the T_0_ group. Similar studies reported by Li et al. (2019) and Saranya et al. (2015) argued that curcumin relieved liver damage induced by AFB1 by decreasing AFB1-DNA adducts in the liver [28,35].

The expression levels of genes related to cytochrome P450s in healthy individual are lower than those in specimens stimulated by exogenous chemicals [36]. Some studies showed that genes expression related to CYP450 in tissues was modulated by nutritional factors in turkeys and chicken and inhibited by polyphenols in humans [9,37]. The results of this study demonstrated that CYP450 protein content was significantly increased in injured liver after AFB1 administration; there was a significant decrease in CYP450 protein content in the T_500_ + AFB1 group. No significant increase in CYP450 content in the T_500_ +AFB1 group was observed when compared with the T_0_ group. Limaye et al. (2018) reported a similar report, arguing that curcumin inhibited hepatic activation of AFB1 to toxic metabolic forms by decreasing the generation of CYP450 [38].

Previous studies demonstrated that curcumin inhibited the hepatic activities in CYP3A, CYP2D6, CYP1A4, CYP3A4, and CYP2C9 in humans and CYP1A1, CYP1A4, CYP2A6 and CYP3A4 in chicks [7,39]. Herein, in order to investigate expression changes in genes related to CYP450 in the liver of ducks, the gene expression levels containing CYP1A1, CYP1A4, CYP2A6, and CYP3A4 in duck liver were determined. The mRNA levels of CYP1A1, CYP1A4, CYP2A6, and CYP3A4 and related protein contents in CYP1A1 and CYP3A4 were increased in the liver injured by AFB1 administration, which is consistent with a previous study reporting that AFB1 was metabolized by the related cytochrome P450s [40]. Dietary curcumin significantly decreased the expression levels of CYP450s (CYP1A1, CYP1A4, CYP2A6, and CYP3A4) in the injured liver in the T_500_ + AFB1 group compared with the T_0_ + AFB1 group. Similar to our report, Pauletto et al. (2020) presented that curcumin supplementation alleviated liver damage by inhibiting the CYP2A6 gene expression in broilers treated with curcumin supplementation and AFB1 administration [8]. In addition, a previous study demonstrated that bush sophora root polysaccharide (BSRPS) eliminated liver injury induced by AFB1 by increasing the SOD2 protein content to inhibit CYP1A5 protein levels, which supported the research results whereby the upregulation of SOD gene expression significantly inhibited CYP450 activity in injured liver after AFB1 administration [40].

Oxidation stress can cause a great harm to many important physiological functions in livestock and poultry, such as liver function, renal function and immunity function, et al. T-AOC, CAT, SOD, GSH, and GST play a vital role in maintaining the capacity of the cellular antioxidant defense system, which could alleviate oxidation stress [41,42,43,44]. The decrease in antioxidant enzymes activity and the increase in MDA and H_2_O_2_ content could lead to an imbalance between oxidation and antioxidants in the body. In this study, AFB1 administration induced oxidative stress, indicating that a decrease in antioxidant activities (T-AOC, CAT, and T-SOD) and GSH content, and an increase in MDA and H_2_O_2_ content in the liver. Notably, adding curcumin into the diet diminished these negative effects induced by AFB1, which is in line with a study reported by Wang et al. (2018) [45]. Changes in these antioxidant enzymes activities containing T-AOC, CAT, and T-SOD, and contents in GSH, MDA, and H_2_O_2_ in the liver in this study indicated that adding curcumin into the diet attenuated the damage to antioxidant defense systems in the damage liver induced by AFB1 administration, which attributed to the properties of curcumin of scavenging free radicals, inhibited oxidative enzymes and lipid peroxidation, and restored the antioxidant status [46]. In addition, AFB1 administration significantly decreased the GST activity, which in line with a previous study [47]; however, adding curcumin into the diet restored GST activity in duck liver, which may be related to the activation of the Nrf2 signaling pathway. GSH plays an important role in maintaining the normal structure and function of cells via the antioxidant system of redox and detoxification reaction, which is another key detoxification cofactor of GST for AFB1 [48]. In this study, there was a significant decrease in GSH values in the liver of ducks after AFB1 administration. As expected, dietary curcumin improved GSH level in ducks, which may relate to the fact that curcumin improves gene expression of glutamate-cysteine ligase (GCL), then induces de novo synthesis of GSH and elevates the level in cellular GSH [49]. Our results demonstrated that curcumin protect liver against oxidative stress induced by AFB1, which is in line with previous studies that curcumin has an ability to alleviate oxidative stress in rats induced by AFB1 administration in rats [21,50]. This study reports that curcumin may have an ability to alleviate oxidative stress induced by AFB1.

The Nrf2-ARE signaling pathway is crucial for the body in regulating oxidative stress. Nrf2 has the ability to diminish oxidative stress in injured liver. When dissociated from the Keap1-Nrf2 complexity in the cytoplasm, Nrf2 is translated into the nucleus and bound to the antioxidant response element (ARE) and upregulates the expression of downstream genes [51,52]. Nrf2 regulates gene expression of both antioxidant genes (CAT, SOD1, GPX1, and GST) and phase (II) detoxifying enzyme genes (NQO1, HO-1, GCLC, and GCLM) [53]. In this study, the mRNA expressions in Nrf2 gene and a series of downstream genes including antioxidant genes (CAT, SOD1, GPX1, and GST) and phase (II) detoxifying enzyme (NQO1, HO-1, GCLC, and GCLM), were inhibited in the liver of ducks by AFB1 administration; in addition, curcumin supplementation significantly altered these genes expression. Overall, this study reported that dietary curcumin protected liver against damage and oxidative stress induced by AFB1 administration by regulating Nrf2-ARE signaling pathway to enhance the antioxidant ability in liver of ducks. A similar study shown that dietary curcumin significantly increased genes expression containing HO−1, Cu/ZnSOD, CAT, γ-GCLC, γ-GCLM, and GPx via the activation of the Nrf2 signaling pathway to enhance the resistant in broiler to heat stress [54]. Jin et al. (2021) also reported that curcumin supplementation alleviated the oxidation stress in the ileum of ducks induced by AFB1 by activating Nrf2 signaling pathway [55]. Overall, their results support our results, in that curcumin has the ability to alleviate oxidative stress induced by AFB1 via the Nrf2 signaling pathway.

The NLRP3–caspase-1 signaling pathway is a typical signaling pathway that mediates inflammatory response. NLRP3 inflammasome could be activated by oxidative stress [56]. ROS are generally generated by redox potent responses and can activate the mitochondrial electron transport chain (ETC) and induce tissue injury [57]. The activation for ROS leads to dissociation of thioredoxin-interacting protein (TXNIP) from oxidized thioredoxin-1 (Trx-1). Trx-1 activates the NLRP3 pathway via association with TXNIP [58], and then activates caspase-1 to accelerate the production of proinflammatory cytokines IL-1β/IL-18. Inflammation inhibition is another mode of curcumin action to protect the liver against injury [59]. Gong et al. (2015) reported that curcumin has the ability to inhibit NLRP3 inflammation and IL-1β content induced by LPS, essentially due to its anti-inflammatory and anti-oxidative properties [18]. In addition, similar studies showed that curcumin inhibited NLRP3 protein expression, caspase1-p20 activation, and caspase-1 and IL-1β levels in lupus-prone mice, as well as suppressed NLRP3 inflammation and IL-1β level in rats [17,55,60]. This supports the results of this study, in that AFB1 administration significantly increased gene and (or) protein expression of TXNIP, NLRP3, caspase-1, and IL-18 in the NLRP3–caspase-1 signaling pathway, which may be related to the oxidative stress induced by AFB1 administration. However, adding curcumin into the diet inhibited related gene expression in the NLRP3–caspase-1 signaling pathway in this assay, which is in line with our previous report arguing that curcumin supplementation could suppress the inflammatory cytokines production induced by AFB1 in duck ileum [55]. Overall, these previous results support our results in this study, in that curcumin relieved inflammation and liver damage induced by AFB1 via inhibiting the NLRP3–caspase-1 signaling pathway.

## 5. Conclusions

**Figure 9:**
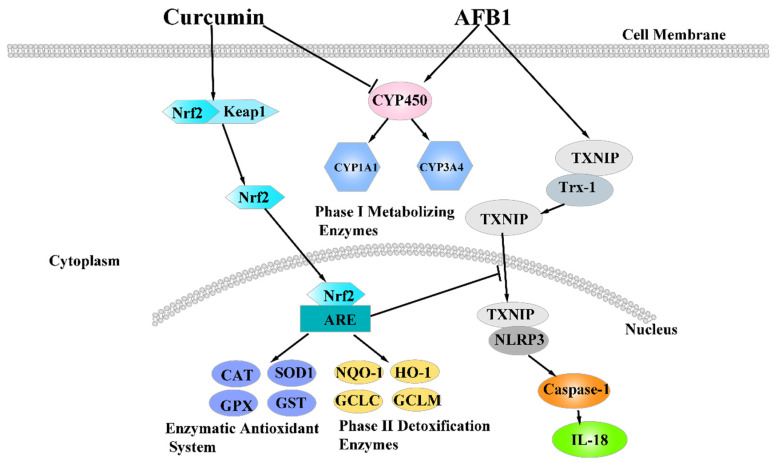
The mechanism of dietary curcumin alleviated liver damage induced by AFB1.

In the present study, curcumin supplementation ameliorated AFB1 induced acute liver lesion, detoxification, oxidative stress, and inflammation, strengthened GST-mediated detoxification; and decreased the generation of CYP450 and AFB1-DNA adducts in liver. Moreover, curcumin supplementation ameliorated acute liver lesion induced by AFB1 by inhibiting NLRP3–caspase-1 signaling pathway (Figure 9). The results of this study demonstrate that curcumin was an effective feed additive to suppress liver injury induced by AFB1, providing a potential guarantee for production safety and reduction in economic losses induced by AFB1 contamination in the poultry breeding industry.

## Data Availability

The data used and/or analyzed in this study are available from the corresponding author on reasonable request.

## Supplementary Materials

The following are available online at https://www.mdpi.com/article/10.3390/foods10123086/s1, Table S1 shows the experiment design; Table S2 shows the ingredient composition and nutrient content of the basal diet (%, as-fed basis); Table S3 shows the accession number, Primer sequence, and product size of target genes.

## Abbreviations

AFB1	Aflatoxin B1
TP	total protein
ALB	albumin
GLB	globulin
A/G	ALB/GLB
ALT	alanine aminotransferase
AST	aspartate aminotransferase
ALP	alkaline phosphatase
TBIL	total bilirubin
T-AOC	total antioxidant capacity
CAT	catalase
T-SOD	total superoxide dismutase
GSH	reductive glutathione
GST	glutathione S-transferase
H_2_O_2_	hydrogen peroxide
MDA	methane dicarboxylic aldehyde
GSH-Px/GPx	glutathione peroxidase
Keap1	Kelch-like ECH-associated protein
Nrf2	nuclear factor erythroid 2-related factor 2
NQO1	NAD(P)H quinone oxidoreductase 1
HO-1	heme oxygenase 1
GCLC	glutamate cysteine ligase catalyzes subunits
GCLM	glutamic acid cysteine ligase modified subunit
TXNIP	thioredoxin interacting protein
NLRP3	NOD-like receptor family pyrin domain containing protein 3
Caspase-1	cysteine-dependent aspartate-directed protease-1
CYP1A1	cytochrome P450 1A1
CYP1A4	cytochrome P450 1A2
CYP2A6	cytochrome P450 2A6
CYP3A4	cytochrome P450 3A4
