# Supplementary material for: Dietary Curcumin Alleviated Aflatoxin B1-Induced Acute Liver Damage in Ducks by Regulating NLRP3–Caspase-1 Signaling Pathways
Source: Foods. 2021 Dec 13;10(12):3086. doi: 10.3390/foods10123086 (PMC8701407; doi:10.3390/foods10123086)
Supplement: Supplementary file 1 [file foods-10-03086-s001.zip › foods-1451171-supplementary.pdf]

Table S1 Experiment design.

Groups	Basal diet	Curcumin (mg curcumin /kg basal diet)
T0	corn-soybean	0
T0 +AFB1	corn-soybean	0
T500+AFB1	corn-soybean	500

T_0: ducks fed basal diet for 70 days then fed PBS water on 70 day; T_0 +AFB1: ducks fed basal diet for 70 days then fed 60 μg of AFB1/kg of duck body weight on 70 day; T_{500} +AFB1: ducks fed 500 mg of curcumin kg^{-1} of basal diet for 70 days then fed 60 μg of AFB1 kg^{-1} of duck body weight on 70 day.

Table S2 Ingredient composition and nutrient content of the basal diet (%, as-fed basis).

Items	1-4 weeks	5-8 weeks	9-10 weeks
Ingredient			
Corn (7.9)	61.70	68.94	75.80
Soybean meal (45)	26.09	26.80	20.10
Corn protein flour (55)	7.90	—	—
Dicalcium phosphate	1.40	1.40	1.40
Limestone	1.08	1.06	1.06
Salt	0.38	0.38	0.38
DL-Methionine	0.15	0.22	0.16
L-Lysine	0.20	0.10	0.00
choline chloride (50%)	0.10	0.10	0.10
Premix	1.00[1]	1.00[2]	1.00[3]
Total	100	100	100
Nutritional level			
Calculated nutrient [4]			
Metabolic energy (MJ/kg)	12.14	11.98	12.21
CP (%)	20.67	17.51	15.03
Calcium (%)	0.90	0.90	0.88
Total phosphorus (%)	0.68	0.67	0.65
Non-phytate phosphorus (%)	0.44	0.44	0.44
Lysine (%)	1.07	0.95	0.71
Methionine (%)	0.48	0.48	0.39
Methionine +cystine (%)	0.81	0.75	0.63
Threonine (%)	0.75	0.66	0.56
Tryptophane (%)	0.21	0.19	0.16

1 The premix provided per kilogram diet: vitamin A 4000 IU, vitamin D3 2000 IU, vitamin E 20 mg, vitamin K3 2.0 mg, vitamin B1 2.0 mg, vitamin B2 12 mg, vitamin B6 3.0 mg, vitamin B12 0.02 mg, nicotinic acid 50 mg, D-pantothenic acid 10 mg, folic acid 1 mg, biotin 0.2 mg, Cu 8 mg, Fe 60 mg, Mn 100 mg, Zn 60 mg, Se 0.2 mg, I 0.4 mg.

2 The premix provided per kilogram diet: vitamin A 3000 IU, vitamin D3 2000 IU, vitamin E 10 mg, vitamin K3 2.0 mg, vitamin B1 1.5 mg, vitamin B2 8 mg, nicotinic acid 30 mg, D-pantothenic acid 10 mg, vitamin B6 3.0 mg, vitamin B12 0.02 mg, biotin 0.1 mg, folic acid 1 mg, Cu 8 mg, Fe 60 mg, Mn 80 mg, Zn 40 mg, Se 0.2 mg, I 0.4 mg.

3 The premix provided per kilogram diet: vitamin A 2500 IU, vitamin D3 1000 IU, vitamin E 10 mg, vitamin K3 2.0 mg, vitamin B1 1.5 mg, vitamin B2 8 mg, nicotinic acid 30 mg, D-pantothenic acid 10 mg, vitamin B6 3.0 mg, vitamin B12 0.02 mg, biotin 0.1 mg, folic acid 1 mg, Cu 8 mg, Fe 60 mg, Mn 80 mg, Zn 40 mg, Se 0.2 mg, I 0.3 mg.

4 Values were calculated based on the data provided by Feed Database in China (2004).

Table S3 Accession number, Primer sequence, and product size of target genes

Transcripts	Accession number		Sequence (5'-3')	Product Length（bp）
Keap1	MF774811.1	Forward	TCACCCTCCATAAACCCACCCAAG	102
		Reverse	AGTAGCCCAAGGACTGCCGATAG	102
Nrf2	NM_001310777.1	Forward	GTTGAATCATCTGCCTGTGG	171
		Reverse	TAAGCTAGGTGGTCGAGTGC	172
HO-1	KU048806.1	Forward	AAGAGCCAGGAGAACGGTCACC	139
		Reverse	TGCCCACCAGGTCTGTCTGAC	139
SOD1	XM_013097859.1	Forward	CCTGTGGTGTCATCGGAATA	116
		Reverse	TTGAACGAGGAAGAGCAAGTA	127
GCLC	XM_027455104.1	Forward	TTCAGGTGACATTCCAGGCTTGC	108
		Reverse	AGAACGGAGATGCAGCACTCAATG	108
GCLM	XM_027462629.1	Forward	TGTTGTGTGATGCCACCTGATCTC	150
		Reverse	CCATTCGTGTGCTTTGACGTTCTG	150
CYP1A1	NM_205147.1	Forward	AGGACGGAGGCTGACAAGGTG	104
		Reverse	AGGATGGTGGTGAGGAAGAGGAAG	104
CYP1A4	XM_027466425.1	Forward	CCACGCAGATCCCAAACGAG	120
		Reverse	TGTGAGGGTACGTCACGAGG	120
CYP2A6	KX687985.1	Forward	CAGGCCCTCTCCTAAACAGATG	81
		Reverse	AATGCAAACGGCACCTTCAGA	81
CYP3A4	XM_015294357.2	Forward	GGCAGCCTGTGATGGCTATT	95
		Reverse	ACCAAAGACCCTGCGATTGG	95
CAT	KU048802.1	Forward	TGTGCGTGACTGACAACCAAGG	96
		Reverse	ACATGCGGCTCTCCTTCACAAC	96
NQO-1	XM_027466610.1	Forward	CGTCGCCGAGCAGAAGAAGATC	195
		Reverse	CTGGTGGTGAACGACAGCATGG	195

NLRP3	MH373356.1	Forward	CGCTGAACGAGGACGCACTG	124
		Reverse	TGGAAGGGTAGTCGGGACATAGC	124
TXNIP	XM_032204531.1	Forward	GCTGCCAAGAAGGAGAAGAAGGTG	130
		Reverse	TGTTCTCGAAGTCGGCGTTGATG	130
Caspase-1	XM_027446016.1	Forward	GCGGAACCAAGAGCAGAGATGAG	130
		Reverse	CCACGGCAGGACTGGATAATAACC	130
IL-18	NM_001310420.1	Forward	GGCTCTGTCCCAAGGCAGGAG	124
		Reverse	GCCACTCTGCGTCAGCTTCAC	124
GST	LOC101797566	Forward	ACAAGGCTGCAACCAGATACTTCC	178
		Reverse	ACTGCACATCTGCTCTGCTAAGC	178
GPX	XM_027459004.1	Forward	GAACGGCACCAACGAGGAGATC	99
		Reverse	TTCACCTGGCACTTCTGGAACAG	99
β-actin	EF667345.1	Forward	ATGTCGCCCTGGATTTCG	62
		Reverse	CACAGGACTCCATACCCAAGAA	62
